# Commentary on “MelanomaDB: a web tool for integrative analysis of melanoma genomic information to identify disease-associated molecular pathways”

**DOI:** 10.3389/fgene.2013.00156

**Published:** 2013-08-13

**Authors:** William C. Reinhold

**Affiliations:** Laboratory of Molecular Pharmacology, Center for Cancer Research, National Cancer Institute, National Institutes of HealthBethesda, MD, USA

In the recent manuscript “MelanomaDB: a web tool for integrative analysis of melanoma genomic information to identify disease-associated molecular pathways” (Trevarton et al., 2013) an interesting dichotomy presents itself, which is in fact more broadly applicable to the field in general. In this work, the authors introduce an integrative tool designed to facilitate and organize disparate forms of data relevant to melanoma, including sequence, microarray, biological, drug target, drug-ability, biomarker, pharmacological, clinical trial, survival, and pathway information. It combines this data into a single matrix, for the purpose of facilitating gene set analysis interpretation, rational experimental design, interpretation of molecular profiles of tumors for individual patients, and aiding in patient stratification.

Included in their tool currently or prospectively are data from the DrugBank^1^, KEGG Drug^2^, the Therapeutic Targets Database^3^, ClinicalTrials.gov^4^, KEGG BRITE^5^, DrugEBIlity^6^, UniProt^7^, the Secreted Protein Database^8^, KinBase^9^, Gene Expression Omnibus (GEO)^10^, Cancer Cell Line Encyclopedia^11^, the Catalogue Of Somatic Mutations In Cancer (COSMIC)^12^, Matched Pair Cancer Cell Lines^13^, Australia's Melanoma Genome Project^14^, The Cancer Genome Atlas (TCGA) project^15^, Oncomine^16^, the Broad Institute's Melanoma Genomics Portal^17^, as well as data from multiple publications.

Certainly this may be seen as an asset. No one group has the ability to generate all the data needed for true systems biology or pharmacology, and so, as a field we are all dependent on data generated by others. The MelanomaDB tool brings together multiple forms of data that, while available from their individual sources, would be challenging, time consuming, and require specific knowledge of those multiple data sources for the user to compile. Especially of interest is the integration of the molecular forms of gene data with those genes commonly mutated in metastatic melanomas, and drug-ability information. Thus, the authors aim to facilitate the fluent integration of disease-relevant information, a huge problem in the field in general.

Unfortunately, there are also inherent dangers for this type of approach. An obvious danger is that when compiling data from multiple sources, one will be subject to any flaws inherent in those data. That is, one is heavily reliant on the work of other groups that one has no detailed knowledge of. Assessment of the reliability of the component parts that are being assembled from multiple data sources is difficult or impossible. Nonetheless, all conclusions are completely reliant on these data. Websites that integrate data from other websites clearly are susceptible to perpetuating data problems or inaccuracies as well as potentially amplifying their influence in the field.

Some forms of data will be more problematic than others. DNA sequence and copy number should be relatively consistent, due to DNAs stability, reproducibility, and ease of verification. The drug databases will give an accurate picture of the incomplete knowledge of the day, realizing that target and interacting pathway information remains incomplete. mRNA and microRNA expression is and will remain subjective due to the technique and reagents used during growth and/or harvest of either cell lines or patient samples. Inclusion of gene set analysis approaches clearly introduces an additional layer of study-specific considerations.

For the DNA sequence data, the ability to repeat analysis provides a way to catch potential errors, however, once erroneous data is entered into a database it will likely remain there. The drug knowledge databases are constantly being updated as new information is obtained. mRNA (or microRNA) expression may be the most difficult to assess, as there is really no way to exactly reproduce another group results, and so there is no clear way to recognize or filter out poorly done studies.

Certainly the MelanomaDB site is not the first to be affected by these considerations, as they are endemic to the field. Careful consideration of one's sources of data, its reliability, and compatibility with other forms of data seems requisite. While recognizing that a detailed assessment of multiple data sources is outside of the scope for this (or any other) group, some consideration of what data to use and its reliability are important if the field is to make accurate and scientifically relevant conclusions. Only by inclusion of high-quality input data may one expect to draw meaningful conclusions.

## Footnotes

^1^DrugBank. http://www.drugbank.ca

^2^KEGGDrug. http://www.genome.jp/kegg/drug/

^3^Therapeutic Targets Database. http://bidd.nus.edu.sg/group/cjttd/

^4^ClinicalTrials.gov. http://clinicaltrials.gov

^5^KEGGBRITE. http://www.genome.jp/kegg/brite.html

^6^DrugEBIlity. (http://www.ebi.ac.uk/chembl/drugebility/)

^7^UniProt. http://www.uniprot.org

^8^Secreted Protein Database. http://spd.cbi.pku.edu.cn

^9^KinBase. http://kinase.com/kinbase/

^10^Gene Expression Omnibus. http://www.ncbi.nlm.nih.gov/geo/

^11^Cancer Cell Line Encyclopedia. http://www.broadinstitute.org/software/cprg/?q=node/11

^12^Catalogue Of Somatic Mutations In Cancer. http://cancer.sanger.ac.uk/cancergenome/projects/cosmic/

^13^Matched Pair Cancer Cell Lines. http://www.sanger.ac.uk/genetics/CGP/Studies/Matched/

^14^Australia's Melanoma Genome Project. http://www.melanoma.org.au/research/melanoma-genome-project.html

^15^The Cancer Genome Atlas. http://cancergenome.nih.gov

^16^Oncomine. http://www.oncomine.com/resource/login.html

^17^Broad Institute's Melanoma Genomics Portal. http://www.broadinstitute.org/software/cprg/?q=node/46

## References

[B1] Trevarton A. J. Mann M. B. Knapp C. Araki H. Wren J. D. Stones-Havas S. (2013). MelanomaDB: a web tool for integrative analysis of melanoma genomic information to identify disease-associated molecular pathways. Front. Oncol. 3:184 10.3389/fonc.2013.00184 23875173 PMC3712543

